# sST2 levels and 3D speckle tracking as predictors of CAD severity in chronic coronary syndrome

**DOI:** 10.1186/s43044-024-00588-x

**Published:** 2024-12-16

**Authors:** Amr Setouhi, K. Maghraby, Nasser Taha, M. Abdelsayed, Mohammed H. Hassan, Hossam Eldin M. Mahmoud

**Affiliations:** 1 Department of Cardiology, Faculty of Medicine, Minia University, Minya, Egypt; 2 Cardiology Division of Internal Medicine Department, Faculty of Medicine, South Valley University, Qena, 83523 Egypt; 3 Department of Medical Biochemistry, Faculty of Medicine, South Valley University, Qena, 83523 Egypt

**Keywords:** Plasma soluble suppression of tumorigenicity 2, Speckle-tracking echocardiography, Coronary artery disease, Chronic coronary syndrome, Gensini score

## Abstract

**Background:**

Previous studies on the relation of sST2 with atherosclerotic disease mostly focused on the predictive value of sST2 for heart failure. However, there is no definite conclusion about the correlation between sST2 level and a complex coronary lesion morphology detected with coronary angiography (CAG). The purpose of this work was to know sST2 level and 3D speckle-tracking echocardiography as predictor of coronary artery disease (CAD) severity in chronic coronary syndrome (CCS) individuals using Gensini score. This prospective cohort work was performed on 90 participants aging from 18 to 80 years old, both sexes, with stable angina pectoris. Participants had been categorized into three groups: Group I (*n* = 30): control group scheduled by normal coronary angiography and group II (*n* = 60): case group which subdivided according to Gensini score into two equal subgroups: IIa: simple lesion (Gensini score < 20) and group IIb: complex lesion (Gensini score of ≥ 20). Plasma sST2 levels were measured in all participants using ELISA technique.

**Results:**

GLS, GAS, GCS and ST2 can significantly predict severity of CAD in CCS, respectively (*P* < 0.001 and AUC (95% CI) = 0.949(0.881–0.984), 0.980(0.925 to 0.998), 0.908(0.828 to 0.959) and 0.702(0.597 to 0.794)) at cutoff ≥ − 10, − 21, − 12 and ≥ 10 with 96.67% (82.8% to 99.9%), 96.67% (82.8 to 99.9), 86.67% (69.3 to 96.2) and 63.33% (43.9 to 80.1) sensitivity (95% CI), 76.67% (64.0% to 86.6%), 85.0% (73.4 to 92.9), 73.33% (60.3 to 83.9) and 65.0% (51.6 to 76.9) specificity (95% CI), 67.44%, 76.32%, 61.90% and 47.50% PPV and 97.87%, 98.08%, 91.67% and 78.00%, NPV with accuracy of 83.33%, 88.89%, 77.78% and 64.44%.

**Conclusions:**

sST2 level, GLS, GAS and GCS can significantly predict severity of CAD in CCS.

## Background

The primary etiology of cardiovascular (CV) mortality and morbidity globally is coronary artery disease (CAD) [1]. CAD is mainly brought on by atherosclerosis of coronaries, that begins in the early years of life and advances at different rates based on the inherent tendency of the person, along with lifestyle changes and medical treatments [2–4] .

CAD may be emerged clinically as either an acute or a more persistent yet chronic worsening of manifestaions. Contemporary categorization of CAD by the European Society of Cardiology involves acute (ACS) and chronic coronary syndromes (CCS) [5]. The noninvasive risk assessment of individuals assessed for chest pain, but without an ACS, has the potential to provide novel possibilities for diagnosis, prognosis, therapy adjustments and enhanced clinical outcomes [6].

Echocardiography is the primary cardiological imaging method used for individuals who are suspected to have heart disease. Conventional echocardiography at rest gives little data on the existence and CAD severity among individuals anticipated of having stable angina pectoris [7].

Noninvasive and quantitative evaluation of global and regional myocardial wall motion may be achieved using real-time three-dimensional speckle-tracking echocardiography (3D-STE). Comparative analysis has been conducted between the efficacy of this technology and the magnetic resonance imaging tagging approach [8]. Emerging research has demonstrated that strain and strain rate are more effective than traditional analysis of wall motion and LVEF in evaluating myocardial systolic dysfunction. Therefore, the strain or strain rate has the capacity to be used in the early assessment of the crucial CAD [9].

Currently, the guidelines for heart failure (HF) recommend sST2 as a cardiac biomarker capable of predicting cardiovascular prognosis related with ACS [10–12]. Prior clinical investigations on the correlation between sST2 and atherosclerotic diseases mostly concentrated on the prognostic significance of sST2 for HF and death, primarily in individuals with ischemic heart disease [13]. Furthermore, other research examined the association between level of sST2 and the CAD prognosis [14, 15]. Nevertheless, the correlation between level of sST2 and the intricate anatomy of coronary lesions identified employing coronary angiography (CAG) in individuals with CAD remains uncertain.

The objective of this work was to know the relationship between sST2 level and 3D-STE as predictor of severity of CAD in CCS patients using Gensini score.

## Methods

This prospective cohort work was conducted on 90 participants aging from 18 to 80 years old, both genders. Patients with stable angina pectoris (chronic coronary syndrome) were defined according to recent ESC guidelines [16] as chest pain or discomfort (angina) suspected to be due to myocardial ischemia. The work had been conducted from October 2022 to October 2023 following approval from the Ethics Committee from Minia University Hospitals, Minia, Egypt. All participants provided well-informed written consent.

Criteria for exclusion were patients who had a history of ACS (Prior STEMI, NSTEMI or unstable angina), cardiomyopathy (of any etiology) with LVEF less than 55%, atrial fibrillation (Afib), significant valvular heart disease, poor image quality, acute autoimmune or infectious disorders, malignancies, dysfunction of thyroid and severe renal or hepatic insufficiency.

Participants had been divided into three groups: Group I (*n* = 30): control group that scheduled by normal coronary angiography and group II (*n* = 60): case group which subdivided into two subgroups equally: IIa: case group with simple lesion (Gensini score < 20) and group IIb: case group with complex lesion (Gensini score of ≥ 20).

Each participant underwent comprehensive taking of history, clinical examinations, laboratory tests [full blood picture (CBC), renal and hepatic function tests].

### Soluble suppression of tumorigenicity 2 assay

Blood samples were withdrawn from all participants utilizing tubes containing EDTA. Prompt centrifugation was followed by storage of the separated plasma samples until analysis as done in our previous work [17]. Analysis of plasma sST2 levels was conducted using ELISA assay kits supplied by Elabscience, USA (catalog No.E-EL-H6082) based on sandwich ELISA technique. The assay calibration was carried out following the guidelines provided by the manufacturer, and subsequent data were normalized to a standard curve using microplate ELISA reader (EMR-500, USA).

### Three-dimensional speckle-tracking analysis

Baseline 3D echocardiography had been conducted before coronary angiography using Echo-machine GE Vivid E95 and 4Vc-D probe. Images were obtained from individuals in the left lateral decubitus posture and linked to an ECG using the echocardiography standards. A rate of frame greater than 25 frames per second was maintained for 3D-STE. Automated measurement of LVEF was conducted using 4D auto LVQ. The program autonomously identifies the endocardial boundary of the cavity of LV and computes the volumes of the LV. If the investigator observed the auto endocardial border detection to be incorrect, the left ventricular endocardial borders were altered manually using a point-click approach in a multiplanar layout consisting of three transverse and three apical planes. Then, this was a secondary automated refinement of the detection of boundaries depending on the obtained findings. After evaluating the left ventricular volumes and EF, an automated mapping of the epicardial boundary was shown to determine the specific area of interest needed for measuring left ventricular mass and myocardial deformation. This trace of epicardium was calibrated manually to involve the whole thickness of the left ventricle wall utilizing the same point-click technique. The LV’s global peak systolic strain, GCS, GAS and GLS were identified as the strain parameters.

### Coronary angiography

CAD was identified when any of the primary coronary arteries, such as left main (LM) coronary and left anterior descending (LAD), left circumflex (LCX) coronary and right coronary arteries, were blocked due to occlusion above 50% of the lumen diameter. When the biochemical condition of a participant is unclear, skilled cardiologists may utilize the score of Gensini to evaluate the CAD severity [18].

### Gensini score

The score of Gensini [19] was measured by multiplying the severity coefficient, that was allocated to each stenosis of coronaries depending on the degree of constricting of the vessel (decreases of 25%, 50%, 75%, 90%, 99% and total blockage were assigned scores of Gensini of 1, 2, 4, 8, 16 and 32, correspondingly), by the coefficient that was established by the functional importance of the myocardial region supplied by this particular segment. This was done in order to arrive at the Gensini score. The LM coronary artery, 5; the LAD coronary artery proximal segment, 2.5; the LAD coronary artery mid-segment, 1.5; the LAD coronary artery apical segment, 1; the 1st diagonal branch, 1; the 2nd diagonal branch, 0.5; the CX artery proximal portion, 2.5 (if Lt coronary artery dominancy exist 3.5); the CX artery distal portion, 1 (if dominant, 2); the obtuse marginal branch, 1; the posterolateral branch, 0.5; the Rt coronary artery proximal portion, 1; the Rt coronary artery mid-segment, 1; the Rt coronary artery distal portion, 1; and the posterior descending artery, 1. A majority of studies classified a score of Gensini ≥ 20 as severe atherosclerosis.

### Sample size calculation

The sample size calculation was performed using EpI-Info 2002 software statistical package designed by World Health Organization (WHO) and by Centres for Disease Control and Prevention (CDC).

The sample size was calculated based on the following considerations: 95% confidence level and the prevalence of accuracy of GLS for prediction of severity of CAD in CCS patients was 90% according to a previous study [20] ± 5% confidence limit. Seven cases were added to overcome dropout. Therefore, we will recruit 90 cases.

### Statistical analysis

Statistical analysis had been carried out utilizing SPSS v26 (IBM Inc., Chicago, IL, USA). Quantitative factors had been displayed as mean and standard deviation (SD) and contrasted across all groups utilizing ANOVA (F) test with post hoc test (Tukey). Qualitative parameters had been displayed as frequencies and percentages (%) and were analyzed employing the Chi-square test. A two-tailed *P* value < 0.05 was deemed statistically significant.

## Ethical considerations

After approval from the Ethical Committee of Faculty of Medicine, Minia University, an Informed written consent was obtained from patients. Adequate provisions to maintain privacy of participants and confidentiality of the data are as follows:

- The patients were given the option of not participating in the study if they did not want to.
- We put code numbers to each participant with the name and address kept in a special file.
- We hid the patients’ names when we used the research.
- We used the results of the study only in a scientific manner and not to use it in any other aims.

## Results

### Demographic data

Age and height were insignificantly different among the three groups. Weight and BMI were insignificantly different between Group IIa and (Group I and Group IIb) and were significantly higher in Group IIb than in Group I (*P* value = 0.017 and 0.007, respectively).

### Risk Factors of the studied groups

DM, hypertension and smoking were significantly different across all groups (*P* < 0.05). Table 1

**Table 1:** Demographic data, patients’ characteristics and risk factors of the groups under the study

		Group I (*n* = 30)	Group IIa (*n* = 30)	Group IIb (*n* = 30)	*P*
Age (years)		52.7 ± 12.56	56.4 ± 12.16	56.2 ± 13.83	0.469
Sex	Male	9(30.0%)	25(83.33%)	24(80.0%)	< 0.001*
	Female	21(70.0%)	5(16.67%)	6(20.0%)	
Weight (kg)		66.2 ± 5.52	71.1 ± 10.51	73.1 ± 11.46	0.019*
		P1 = 0.12, P2 = 0.017*, P3 = 0.696			
Height (m)		1.67 ± 0.08	1.66 ± 0.07	1.65 ± 0.07	0.430
BMI (kg/m)		23.8 ± 3.53	25.7 ± 3.93	27 ± 4.34	0.010*
		P1 = 0.16, P2 = 0.007*, P3 = 0.431			
Risk factors	DM	2(6.67%)	14(46.67%)	23(76.67%)	< 0.001*
	HTN	7(23.33%)	25(83.33%)	23(76.67%)	< 0.001*
	Smoker	4(13.33%)	23(76.67%)	19(63.33%)	< 0.001*

Data are presented as mean ± SD or frequency (%)

*Significant *P* value < 0.05. P1: *P* value between Group I and Group IIa. P2: *P* value between Group I and Group IIb. P3: *P* value between Group IIa and Group IIb

BMI, Body mass index; HTN, hypertension; DM, diabetes mellitus

### Echocardiographic findings

LVEDV, LVESV and LVEF had been significantly various across the three groups (*P* < 0.001). LVEDV and LVESV had been substantially greater in Group IIa and Group IIb in contrasted to Group I (*P* < 0.05) and in Group IIb in contrasted to Group IIa (*P* < 0.05). LVEF was substantially reduced in Group IIa and Group IIb contrasted to in Group I (*P* < 0.05) and in Group IIb contrasted to in Group IIa (*P* < 0.001). Table 2

**Table 2:** 3D Echocardiography of the studied groups

	Group I (*n* = 30)	Group IIa (*n* = 30)	Group IIb (*n* = 30)	*P*
LVESV (mL)	43.3 ± 11.67	58 ± 12.96	70.8 ± 9.08	< 0.001*
	P1 < 0.001*, P2 < 0.001*, P3 < 0.001*			
LVEDV (mL)	91.5 ± 18.47	110.3 ± 18.91	124.7 ± 15.88	< 0.001*
	P1 < 0.001*, P2 < 0.001*, P3 = 0.006*			
LVEF (%)	66.5 ± 3.7	63.7 ± 3.08	60 ± 3.06	< 0.001*
	P1 = 0.004*, P2 < 0.001*, P3 < 0.001*			

Data are presented as mean ± SD or frequency (%)

*Significant *P* value < 0.05. P1: *P* value between Group I and Group IIa. P2: *P* value between Group I and Group IIb. P3: *P* value between Group IIa and Group IIb

LVEDV, left ventricular end-diastolic volume; LVESV, left ventricular end-systolic volume; LVEF, left ventricular ejection fraction

### Speckle tracking and sST2 assay

GLS, GAS, GCS and sST_2_ had been significantly various across the three groups (*P* < 0.001 and 0.025, respectively). GLS and GAS were significantly higher in Group IIa and Group IIb in contrasted to Group I (*P* < 0.001) and in Group IIb in contrasted to Group IIa (*P* < 0.001). GCS was insignificantly various among Group I and Group IIa and had been substantially greater in Group IIb in contrasted to (Group I and Group IIa) (*P* < 0.001). sST_2_ was insignificantly various among Group IIa and (Group I and Group IIb) and was significantly higher in Group IIb compared with Group I (*P* = 0.027) Table 3.

**Table 3:** Gensini score, strain parameters and ST2 of the studied groups

	Group I (*n* = 30)	Group IIa (*n* = 30)	Group IIb (*n* = 30)	*P*
Gensini score	0 ± 0	12.3 ± 3.16	35.6 ± 5.37	P3 < 0.001*
Strain parameters				
GLS (%)	− 14.5 ± 2.43	− 10.8 ± 1.99	− 7.2 ± 1.85	< 0.001*
	P1 < 0.001*, P2 < 0.001*, P3 < 0.001*			
GAS (%)	− 39.3 ± 1.31	− 27.5 ± 7.84	− 14.4 ± 2.59	< 0.001*
	P1 < 0.001*, P2 < 0.001*, P3 < 0.001*			
GCS (%)	− 14.6 ± 2.75	− 14.3 ± 2.93	− 8.9 ± 3.04	< 0.001*
	P1 = 0.933, P2 < 0.001*, P3 < 0.001*			
ST_2_ (ng/ml)	8.6 ± 9.13	9.8 ± 9.51	14.8 ± 8.61	0.025*
	P1 = 0.86, P2 = 0.027*, P3 = 0.095			

Data are presented as mean ± SD

*Significant *P* value < 0.05. P1: *P* value between Group I and Group IIa. P2: *P* value between Group I and Group IIb. P3: *P* value between Group IIa and Group IIb

ST2, suppression of tumorigenicity 2; GLS, global longitudinal strain; GAS, global area strain; GCS, global circumferential strain

#### Univariate and multivariate regression of multiple variables versus severity of coronary artery disease

In univariate regression, hypertension, sex, weight, BMI, DM, hypertension, LV ESV, LVEDV, LVEF, GLS, GAS, GCS and ST2 were independent predictors of severity of coronary artery disease (*P* value < 0.05). In multivariate regression, DM LV ESV, LVEDV, LVEF, GLS, GAS, GCS and ST2 were independent predictors of Gensini score (*P* value < 0.05) while sex, weight, BMI and hypertension were not. Table 4

**Table 4:** Univariate and multivariate regression of multiple variables versus severity of coronary artery disease

	Univariate			Multivariate		
	Odds ratio	95% CI	*P*	Odds ratio	95% CI	*P*
Sex (female)	0.326	0.116–0.915	0.033*	9.725	0.844–111.96	0.068
Weight (kg)	1.047	1.0007–1.096	0.046*	0.982	0.891–1.082	0.721
BMI (kg/m2)	1.147	1.023–1.287	0.018*	1.080	0.833–1.4004	0.558
DM	9.035	3.253–25.09	< 0.001*	22.846	2.290–227.83	0.007*
Hypertension	2.876	1.072–7.709	0.035*	0.331	0.0566–1.939	0.220
Smoker	2.111	0.858–5.192	0.103	–	–	–
LV ESV (mL)	1.139	1.077–1.205	< 0.001*	1.123	1.0404–1.212	0.003*
LVEDV (mL)	1.064	1.034–1.095	< 0.001*	1.085	1.0271–1.146	0.003*
LVEF (%)	0.643	0.531–0.778	< 0.001*	0.624	0.4642–0.839	0.001*
GLS (%)	2.941	1.799–4.806	< 0.001*	3.206	1.2854–7.998	0.012*
GAS (%)	1.850	1.272–2.690	0.001*	2.702	1.1910–6.129	0.017*
GCS (%)	2.033	1.484–2.785	< 0.001*	2.084	1.3933–3.118	< 0.001*
ST2 (ng/ml)	1.066	1.015–1.119	0.009*	1.093	1.0001–1.195	0.049*

CI, confidence interval

*Significant as *P* value ≤ 0.05

#### Role global longitudinal strain, global area strain, global circumferential strain, suppression of tumorigenicity 2 in prediction of severity of coronary artery disease in chronic coronary syndrome.

**Fig. 1:**
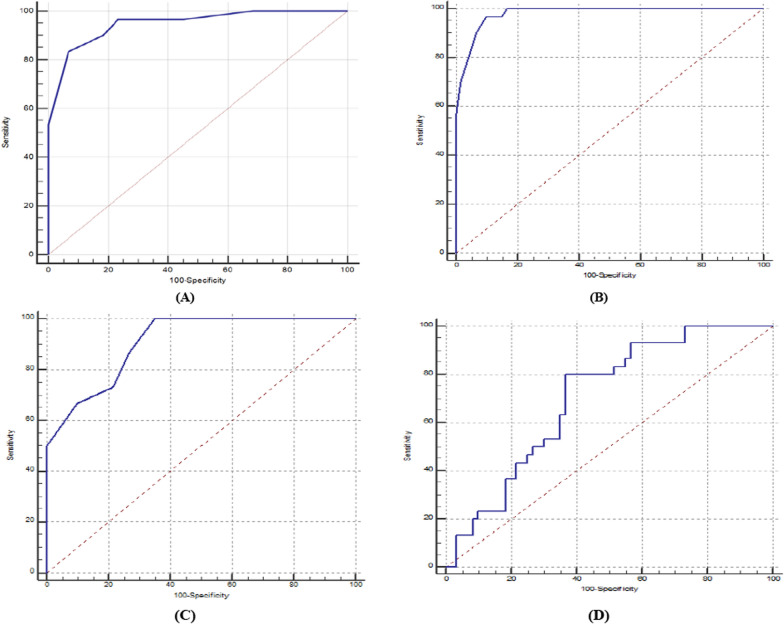
ROC curve of prediction of severity of coronary artery disease in chronic coronary syndrome **A** global longitudinal strain, **B** global area strain, **C** global circumferential strain, **D** suppression of tumorigenicity 2

GLS, GAS, GCS and ST2 can significantly predict severity of CAD in CCS, respectively (*P* < 0.001 and AUC (95% CI) = 0.949(0.881–0.984),0.980(0.925 to 0.998), 0.908(0.828 to 0.959) and 0.702(0.597 to 0.794)) at cutoff ≥ − 10, − 21, − 12 and ≥ 10 with 96.67% (82.8% to 99.9%), 96.67% (82.8 to 99.9), 86.67% (69.3 to 96.2) and 63.33% (43.9 to 80.1) sensitivity (95% CI), 76.67% (64.0% to 86.6%), 85.0% (73.4 to 92.9), 73.33% (60.3 to 83.9) and 65.0% (51.6 to 76.9) specificity (95% CI), 67.44%, 76.32%, 61.90% and 47.50% PPV and 97.87%, 98.08%, 91.67% and 78.00%, NPV with accuracy of 83.33%, 88.89%, 77.78% and 64.44%.(Fig. 1A–D**).**

## Discussion

Our study finds that age and height were insignificantly different among the three groups. These results are in agreement with Kim et al. [21] who showed that in total, 184 subjects, who were followed up 1 year after successful primary PCI for STEMI, were evaluated in this study. The mean age of the subjects was 61.4 ± 11.8 years and the age between three groups was indifferent (*P* = 0.058). However, Alkhateeb et al. [17] showed different result as they noted that age was statistically significantly different between 33 ischemic patients and 30 nonischemic controls as the ischemic group had higher levels of aging (*p* < 0.001).

Our study shows that sex, weight and BMI were significantly different among the three groups (*P* value < 0.05). Also, weight and BMI were insignificantly different between Group IIa and (Group I and Group IIb) and were significantly higher in Group IIb than in Group I (*P* value = 0.017 and 0.007, respectively). These findings are in conglomerate with Alkhateeb et al. [17] who demonstrated that gender was different between 33 ischemic patients and 30 nonischemic controls as a higher proportion of males (*P* value = 0.005) compared with the control group. On the other hand, Alkhateeb et al. [17] disagreed to ours as they found that BMI was indifferent between 33 ischemic patients and 30 nonischemic controls (*P* = 0.44).

Our study reports that DM, hypertension and smoker were significantly higher among the three groups. These outcomes are supported by Goda et al. [22] who showed that a total of 577 patients were studied as HTN and DM was significantly more prevalent among high SYNTAX score group with *p* value 0.0001.

Regarding our results, Gensini score was substantially higher in Group IIb compared with Group IIa. Our results are like outcomes of Charach et al. [23] found that Gensini score correlated with the CAD severity and multivessel disease. Added to that, Wang et al. [24] ascertained what we found they stated that score of Gensini is an efficient tool utilized to assess the CAD severity which showed increase in score with increase in severity of CAD.

Our statistics reveal that GCS, GLS and GAS were substantially different among the three groups. GLS and GAS were substantially greater in Group IIa and Group IIb contrasted to in Group I and in Group IIb contrasted to in Group IIa. GCS was insignificantly varied among Group I and Group IIa and had been substantially higher in Group IIb compared with (Group I and Group IIa). These results are on the same side of Joseph et al. [25] who observed that increase in GLS and GAS was correlated with CAD risk and severity. Additionally, Román-Fernández et al. [26] had the same as they revealed that GCS shows more decrease with increase in severity of CAD.

Our work finds that sST_2_ was varied between the three groups as sST_2_ was insignificantly varied among Group IIa and (Group I and Group IIb) and was significantly varied in Group IIb compared with Group I. In agreement to what we found, Li M et al. [27] showed that a greater sST_2_ level is substantially correlated with long-term MACEs and all-cause mortality in CAD individuals.

Our work reveals that GLS can significantly predict severity of CAD in CCS AUC = 0.949 at cutoff ≥ -10 with 96.67% sensitivity, 76.67% specificity, 67.44% PPV and 97.87% NPV with accuracy of 83.33%. Our results are consistent with Billehaug Norum et al. [28] as they stated that the overall weighted mean GLS was − 17.2% ± 2.6 among CAD vs. − 19.2% ± 2.8 in CAD negative individuals. Mean AUC among four studies to predict CAD ranged from 0.68 to 0.80. Additionally, these results are similar to Alaika et al. [29] who demonstrated that resting GLS by STE showed high specificity (> 90%) and commendable sensitivity (60%) in detecting severe CAD in diabetic patients with unknown CAD.

Our results figure out that GAS can significantly predict severity of CAD in CCS (AUC = 0.980) at cutoff ≥ − 21 with 96.67% sensitivity, 85.00% specificity, 76.32% PPV and 98.08% NPV with accuracy of 88.89%.

Concerning our statistics, GCS can significantly predict severity of CAD in CCS and AUC = 0.908 at cutoff ≥ − 12 with 86.67% sensitivity, 73.33% specificity, 61.90% PPV and 91.67% NPV with accuracy of 77.78%. Onishi et al. [30] supported our results as they found that GCS was a rapid means to get myocardial strain comparable to STE. Further, Li N et al. [31] had similar results as they found that GCS was correlated to severity of CAD.

### Limitations

Limitations of the work involved the relatively small sample size. The study’s single-center design and the possible impact of selection, gender and blinding bias on the findings.

## Conclusions

- Elevated baseline sST2 level is associated with a higher risk of CAD complexity in chronic coronary syndrome.
- GLS, GAS, GRS and GCS can significantly predict severity of CAD in chronic coronary syndrome.
- 3D-Speckle tracking echocardiography has higher sensitivity, specificity, PPV, NPV and accuracy than sST2 in prediction of CAD severity.

## Data Availability

No datasets were generated or analyzed during the current study.

## Abbreviations

sST2: Soluble suppression of tumorigenicity 2
CAD: Coronary artery disease
CCS: Chronic coronary syndrome
GAS: Global area strain
GCS: Global circumferential strain
GLS: Global longitudinal strain
LVEF: Left ventricular ejection fraction
3D-STE: Three-dimensional speckle-tracking echocardiography
CA: Coronary angiography
